# Effect of Inquiry-Based Stress Reduction on Well-being and Views on Risk-Reducing Surgery Among Women With *BRCA* Variants in Israel: A Randomized Clinical Trial

**DOI:** 10.1001/jamanetworkopen.2021.39670

**Published:** 2021-12-28

**Authors:** Carla Landau, Anne Marie Novak, Ariel B. Ganz, Benjamin Rolnik, Eitan Friedman, Shahar Lev-Ari

**Affiliations:** 1 Department of Health Promotion, School of Public Health, Sackler Faculty of Medicine, Tel Aviv University, Tel Aviv, Israel; 2 Department of Genetics, Stanford University School of Medicine, Stanford, California; 3 Suzanne Levy-Gertner Oncogenetics Unit, Sheba Medical Center, Ramat Gan, Israel

## Abstract

**Importance:**

The high risk for breast and ovarian cancers conferred by being a carrier of *BRCA1* or *BRCA2* germline variant can negatively impact physical and psychological well-being. Novel nonpharmacological interventions on well-being in women with *BRCA* variants have rarely been reported.

**Objective:**

To determine the effect of a 12-week inquiry-based stress reduction (IBSR) program on psychological well-being, sleep quality, psychosocial variables, and attitudes toward risk-reducing surgical procedures among women in Israel who carried *BRCA* variants.

**Design, Setting, and Participants:**

This randomized clinical trial had a 12-week intervention period and a 12-week follow-up period. It was conducted between April 1, 2017, and July 31, 2020. Participants were recruited from the Meirav Breast Center at the Sheba Medical Center, Israel, and the intervention was conducted in Tel Aviv, Israel. The cohort included women with *BRCA* variants. Data were analyzed from August 1 to December 1, 2020.

**Interventions:**

Women were randomly assigned to the 12-week IBSR program or standard care. The IBSR technique is based on the skills of mindfulness, inquiry, and cognitive reframing. The intervention included standardized, weekly group meetings conducted throughout 12 weeks. Standard care included semi-annual breast examinations and breast magnetic resonance imaging (alternating), a gynecological examination, a transvaginal ultrasonographic examination, and CA-125 serum determination. Differences between the groups were tested using mixed-effects models in an intent to treat analysis.

**Main Outcomes and Measures:**

The primary outcome was psychological well-being, including 6 parameters: autonomy, personal growth, positive relationships, control of the environment, goals in life, and self-acceptance. Secondary outcomes included sleep quality, attitudes toward risk-reducing surgical procedures, and psychosocial variables. Questionnaires were administered at baseline (T1), at completion of the 12-week intervention (T2), and 12 weeks after completion of the intervention (T3).

**Results:**

Overall, 100 women (mean [SD] age, 41.37 [11.06] years) completed the study, with 50 randomized to the intervention group and 50 randomized to the control group. Mean (SD) time from variant discovery was 4.7 (3.3) years. There were no differences between the intervention and control groups in baseline mean (SD) scores of psychological well-being parameters (autonomy: 55.20 [11.12] vs 56.77 [9.90]; environmental control: 56.30 [11.98 vs 58.51 [11.41]; positive relationships: 63.10 [15.91] vs 68.10 [9.86]; goals in life: 60.00 [14.12] vs 64.82 [10.57]; self-acceptance: 55.02 [16.62] vs 60.32 [13.50]) except personal growth (63.70 [14.66] vs 68.85 [8.07]). The IBSR group, compared with the control group, experienced better mean (SD) scores on all psychological well-being parameters at T2 (autonomy: 63.64 [8.35] vs 54.73 [10.41]; environmental control: 63.95 [10.05] vs 57.45 [11.43]; personal growth: 73.00 [8.34] vs 65.76 [10.95]; positive relationships 71.17 [9.99] vs 65.06 [12.58]; goals in life: 67.57 [8.88] vs 61.18 [12.87]; self-acceptance: 66.93 [11.15] vs 58.09 [15.55]) and at T3 (autonomy: 62.68 [9.05] vs 56.12 [10.64]; environmental control: 64.55 [10.28] vs 59.35 [12.98]; personal growth: 72.00 [8.06] vs 67.15 [11.82]; positive relationships: 71.24 [9.78] vs 66.92 [12.37]; goals in life: 68.33 [8.54] vs 62.92 [13.24]; self-acceptance: 66.84 [11.35] vs 58.97 [17.03]). Individuals in the IBSR group also experienced statistically significant improvements in sleep quality (mean [SD]: T1, 7.35 [3.97]; T3, 4.63 [3.21], *P* < .001), whereas the control group experienced no statistically significant difference. Women in the intervention group had a more favorable consideration of risk-reducing oophorectomy, from 7 women (14%) who refused to consider oophorectomy at T1 to 1 woman (2%) who refused to consider it at T3 (*P* = .04), and similar change in consideration of mastectomy: from 23 women (46%) who refused to consider mastectomy at T1 to 13 women (29%) who refused to consider it at T3 (*P* < .001).

**Conclusions and Relevance:**

This randomized clinical trial found that IBSR improved psychological well-being and led to a more favorable view on risk-reducing surgical procedures for at least 6 months among women in Israel who carried *BRCA* variants. These results suggest that IBSR may be implemented as a self-practice tool to enhance the well-being of individuals who carry *BRCA* variants and support them in their decision-making processes.

**Trial Registration:**

ClinicalTrials.gov Identifier: NCT03162276

## Key Points

**Question:**

Does the inquiry-based stress reduction technique improve the psychological well-being, sleep quality, and psychosocial variables and change views on risk-reducing surgery in women in Israel with *BRCA1*/*BRCA2* variants?

**Findings:**

This randomized clinical trial including 100 patients using an IBSR intervention found that IBSR improved the well-being and sleep quality of women with *BRCA1*/*BRCA2* variants. After the intervention, the participants who underwent IBSR had more favorable view on risk-reducing oophorectomy and mastectomy.

**Meaning:**

These findings suggest that IBSR may be used as a tool to enhance the psychological well-being of women with *BRCA* variants and support them in considering risk-reducing surgery.

## Teaser

This randomized clinical trial assessed the effect of an inquiry-based stress reduction intervention on psychological well-being, sleep quality, and receptivity to risk-reducing surgical procedures in asymptomatic women with *BRCA* variants.

## Introduction

Breast cancer is the most prevalent type of malignant neoplasm in the world, with approximately 2.2 million new cases worldwide in 2020.^1^ Approximately 5% to 10% of breast cancers are hereditary, hallmarked clinically by unusual transgenerational clustering of breast cancers and bilateral and early onset of disease, at times associated with ovarian cancer.^2^ Germline pathogenic variants in the *BRCA1* (OMIM 113705) or *BRCA2* (OMIM 600185) genes underlie most hereditary breast and ovarian cancers.^2,3^ Individuals who carry *BRCA* variants and are asymptomatic are at a substantially higher lifetime risk of developing breast and/or ovarian cancer compared with the general population, with risks increased by 69% to 72% for breast cancer and 17% to 44% for ovarian cancer.^4,5^ Being a carrier of a BRCA variant often impacts physical and psychological well-being: anxiety,^6^ depression,^7,8,9^ distress,^10,11^ poor sleep quality,^12^ sexual inactivity,^13^ fatigue,^12^ endocrine dysfunction,^14^ and imbalanced caloric intake^15^ have been noted in individuals carrying *BRCA* variants, impairing their quality of life and health status.

Genetic counselling for individuals with *BRCA* variants includes genetic test interpretation, risk assessment, education, and offering measures for early detection and risk reduction.^16^ There is no consensus or guidelines on the role of counselors in providing psychosocial support. Psychosocial interventions are defined as “non-pharmacological, involving an interpersonal relationship between an individual or group of individuals, and one or more trained (usually professional) helpers,”^17^ aimed to enhance the psychological and general health outcomes of individuals with a variant.^18^ Previous studies that focused on psychosocial interventions in individuals with *BRCA* variants are few. A recent review found 23 relevant studies published in peer-reviewed journals,^19^ most of which did not have control or comparison groups and measured outcomes qualitatively instead of using validated measures. Except for our own preliminary trial^20^ and telephone peer support,^21^ to our knowledge, no other randomized clinical trial has assessed a nonpharmacological intervention that was found to enhance the psychosocial outcomes among individuals with *BRCA* variants. A 2019 meta-analysis by Jeffers et al^22^ concluded that there was moderate- to very low–certainty evidence on the effects of interventions to improve psychosocial well-being in women with *BRCA* variants after risk-reducing surgical procedures. High levels of psychological well-being parameters are associated with happier lives, a more satisfactory family life, closer relationships, greater probability of engaging in healthy behaviors, general physical health improvements, and lower risk of illness and death in healthy individuals and individuals who are chronically ill.^23,24,25^ It also has been shown that it is possible to improve well-being through a behavioral intervention.^25^

The inquiry-based stress reduction (IBSR) technique is the clinical adaptation of “The Work,” developed by Byron Katie.^26^ The approach is based on the skills and principles of mindfulness and self-inquiry, as applied to cognitive reframing and behavioral change. Several studies have shown that in the general population, practicing the IBSR technique was associated with improved psychopathologic symptoms,^27^ happiness, and perceived quality of life.^28^ Additionally, it reduced occupational burnout in teachers^29,30^ and improved subjective and psychological well-being of teachers during the COVID-19 pandemic.^31^ The benefits of the IBSR technique have been evaluated in individuals with *BRCA* variants^20^ and survivors of breast cancer,^32^ showing improved psychological and physical variables and functional capabilities after completion of treatments.

Shapiro et al^33^ suggested that the practice of cultivating mindfulness may affect processes of self-regulation, values clarification, cognitive-behavioral flexibility, and exposure, which may lead to a significant shift in perspective (ie, reperceiving). We have previously reported that practice of IBSR was associated with increased perception of autonomy, environmental mastery, and competence among women with *BRCA* variants, demonstrated by women’s ability to put their needs in the center and to attend to them, to manage their surroundings according to their personal needs, and, ultimately, to enhance control over their life.^28^ Therefore, we hypothesized that IBSR may enable participants to gain a sense of control over their health and take concrete steps to adhere to recommendations regarding risk-reducing surgical procedures. This randomized clinical trial assessed the effect of an IBSR intervention on psychological well-being, sleep quality, and patient receptivity to risk-reducing surgical procedures in asymptomatic women with *BRCA* variants.

## Methods

This randomized clinical trial was approved by the ethics committees of the Sheba Medical Center and Tel Aviv University. All participants signed an informed consent form. This study is reported following the Consolidated Standards of Reporting Trials (CONSORT) reporting guideline. This study was conducted between April 1, 2017, and July 31, 2020.

### Study Population and Recruitment Process

Participants were recruited between March 2017 and December 2019 from the Meirav Breast Center at the Sheba Medical Center, Israel. Letters inviting all eligible women describing the study goals, research process, and the intervention were sent out. The eligibility criteria were women, aged 25 to 70 years, no personal history of cancer, and harboring pathogenic variants in *BRCA1* and/or *BRCA2* genes. The data on health and *BRCA* status were collected from the medical records of the participants. Consenting participants were randomly allocated to either the IBSR intervention group or to the control group, using Excel 2019 spreadsheet software version 16.0 (Microsoft) randomization tool at a 1:1 ratio.

Each participant was informed of their allocation automatically after completion of the baseline questionnaire. All women with *BRCA1*/*BRCA2* variants who participated in this study were offered an identical surveillance scheme that was not altered throughout the intervention. Surveillance included a semiannual physician-guided breast examination and breast imaging (alternating mammograms and magnetic resonance imaging, so that every 6 months there is 1 type of breast imaging; a gynecological examination; a transvaginal ultrasonographic examination; and CA-125 serum determination), as outlined previously.^34^ Clinicians were blinded to the allocation of participants to either the treatment or control group. The study flowchart is presented in the Figure. The trial protocol and statistical analysis plan are presented in Supplement 1.

**Figure.**
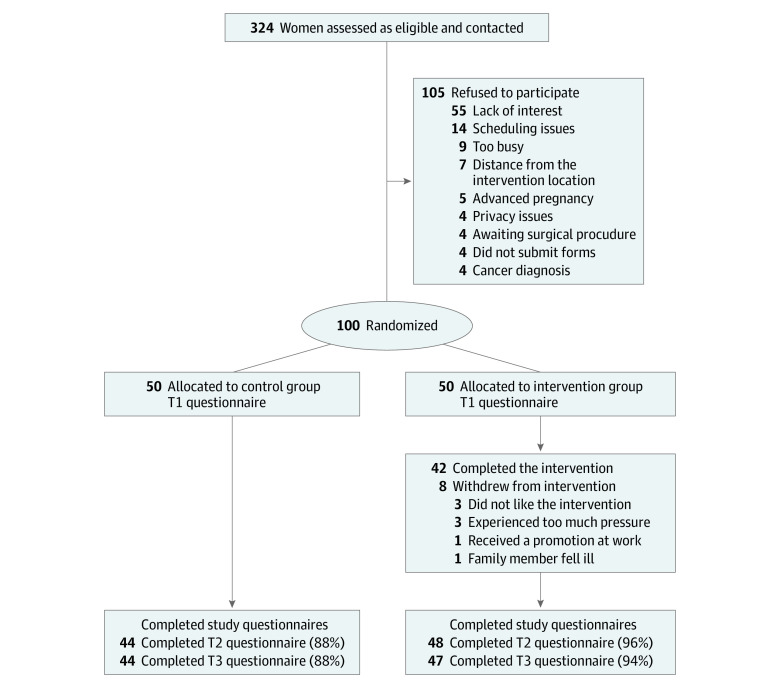
Participant Recruitment Flowchart T1 indicates baseline; T2, immediately after the intervention; T3, 12-week follow-up.

### Study Outcomes

All participants (IBSR and control groups) completed questionnaires that assessed their psychological well-being parameters at 3 time points: the beginning of the intervention (T1; 2 weeks before the intervention to the beginning of the intervention), immediately after the intervention (T2; weeks 12-14), and during the follow-up period (T3; weeks 24-26). The primary outcome measure was psychological well-being, quantified using the Ryff questionnaire (84-item).^23^ The Ryff questionnaire includes 6 subscales that evaluate self-acceptance, positive relationships with others, autonomy, control of the environment, personal growth, and goals in life. For each of the dimensions, the score ranges from 14 to 84. Higher scores indicate higher psychological well-being. Additional parameters included sleep quality, psychosocial variables, and health behaviors. Sleep quality was assessed using the Pittsburgh Sleep Quality Inventory Questionnaire,^35^ which includes 19-items (score range, 0-21) for self-reporting of sleep quality over 1 month; scores of 5 or greater indicate poor sleep quality. Psychosocial variables were positive and negative emotions, measured using Positive and Negative Affect Scales,^36^ a 20-item questionnaire (score range, 10-50 for each subscale) that measures the emotional positive and negative state of individuals; life satisfaction, assessed using the Satisfaction With Life Scale^37^ 5-item scale (score range, 7-35) that measures global life satisfaction; self-assessed health,^38^ a single question designed to assess how the participant evaluates their health; social support, assess using the Perceived Social Support from Family,^39^ a 20-item scale (score range 0-20) that assess the family support as perceived by the respondents; mindfulness, measured using the Mindful Attention Awareness Scale,^40^ a 15-item scale (score range, 15-90) that measures level of attention and mindfulness to the present; optimism, assessed using the Life Orientation Test-Revised^41^ 10-item questionnaire (score range, 0-24) that measures optimism as a character trait, expected to remain stable throughout one’s lifetime; and general self-ability, measured using the General Self-Efficacy^42^ 10-item scale that measures the general perception of personal agency to forecast the individual’s ability to cope with everyday distresses and assess their adaptation to stressful events. Health behaviors were evaluated by the attitudes toward and actual performance of risk-reduction surgical procedures of the breasts and/or fallopian tubes and ovaries.^43^ All outcome measures were validated. Good internal consistency was revealed (α ranged between 0.73-0.93) (eMethods in Supplement 2), indicating a relatively high degree of probability that the items within each instrument were addressing the same constructs.

### Intervention

The 12-week IBSR program is a clinical intervention based on “The Work” method, developed by Byron Katie and run in the US and Europe for 35 years.^26,44^ The IBSR intervention involved weekly group meetings (3 hours/meeting) throughout 12 weeks. Home practice between sessions was supported by facilitator assistants (1-hour session per week). All sessions were standardized according to the IBSR certification program guidelines and assessed to maintain consistency in the program, as described elsewhere.^45^ The intervention program was conducted in 4 groups, with 12 to 18 participants in each group. The meetings were guided by facilitators who were trained in the authorized certification program at The Institute of the Work, an international learning center based in the US.^46^

### Control

Participants in the control group continued standard care as suggested at the Meirav Breast Center, Sheba Medical Center, and completed the measurement tool analyses as the intervention group at 3 points in time, T1, T2, and T3. They received a book on IBSR at the completion of the study.

### Statistical Analysis

The sample size calculation was based on the expected mean (SD) change of 5.74 (9.70)^47^ in any subscale of psychological well-being, the main outcome of this study, using WinPepi software version 11.65 (J. Abramson). A required sample size of 90 participants was calculated to achieve an α of 5% and 80% power. To allow for dropouts and nonattendance (expected 30%), we planned to recruit 118 women. Owing to high response rates of the study (88% in the control group and 94% in the intervention group, defined as completion of all 3 questionnaires of the study), we stopped the recruitment process on reaching 100 participants, an adequate number to enable the required statistical power.

An intention-to-treat analysis approach was implemented for this trial, and all participants were included in the data processing. The similarity of baseline (T1) demographic characteristics of participants in the control and intervention groups was assessed using the χ^2^ test for categorical variables and independent samples *t* test for continuous variables. To ensure the appropriateness of our analysis, we plotted distributions for all measures and changed scores. For most variables, scale values and change scores generally approximated a normal distribution with skewness and kurtosis below 1. Differences between the groups were tested using mixed-model analysis over the 3 different time points as well as the interaction between group and time. The dependent variables were the study outcomes, and the independent variables were time (T1, T2, and T3), group (IBSR and control), and interaction between the time × group. The difference between the outcome variables was measured using the entry values (T1) and the final values gathered at the end of the follow-up (T3). Sensitivity analysis of mixed-model analysis with age was conducted owing to the differences in baseline data between the groups. Control for multiple comparisons was done using the false discovery rate approach.^48^ An analysis of the research hypotheses of the categorical variables and variables without normal distribution (self-assessed health and health behaviors) was performed according to the general estimated equation. To check the internal consistency of the research tools, an α Cronbach coefficient test^49^ was performed for each tool. All data were processed independently by 2 of us (S.L.-A. and E.F.) using SPSS statistical software version 26 (IBM). *P* values were 2-sided, and statistical significance was set at *P* = .05. Data were analyzed from August 1 to December 1, 2020.

## Results

In total, 324 women were contacted and offered participation, of whom 100 signed the informed consent and entered the study, with 50 randomized to the IBSR group and 50 randomized to the control group. The mean (SD) age was 41.37 (11.06) years. Of these, 91 participants (91%; 46 participants in the IRSB group and 45 participants in the control group) completed the study and completed all questionnaires at all 3 of the designated time points. The Figure shows the participant recruitment flowchart of dispersion and the responsiveness of the participants throughout the trial process. Table 1 presents the demographic characteristics of participants. The mean (SD) time from variant discovery was 4.7 (3.32) years. There were no significant between-group differences found at baseline for any of the scales except for mean (SD) age (IBSR: 44.06 [13.05] years; control: 38.68 [7.87] years). Hence, an age-sensitive analysis was conducted. There were no significant between-group differences between participants who completed the study and those who dropped out.

**Table 1.** Demographic Characteristics of Participants

Characteristic	Participants, No. (%)		
	Total	IBSR group	Control group
Country of birth			
Israel	91 (91)	46 (92)	45 (90)
Other^a^	9 (9)	4 (8)	5 (10)
Family status			
Married or in relationship	79 (79)	26 (52)	30 (60)
Not in a relationship^b^	21 (21)	24 (48)	20 (40)
Children^c^			
Yes	69 (70)	36 (73)	33 (66)
No	30 (30)	13 (27)	17 (34)
Education			
High school	14 (14)	8 (16)	6 (12)
College degree	73 (73)	34 (68)	39 (78)
Other higher education	13 (13)	8 (16)	5 (10)
Religious beliefs^d^			
Religious	9 (9)	6 (12)	3 (6)
Traditional	12 (12)	3 (6)	9 (18)
Secular	79 (79)	41 (82)	38 (76)
Variant type^e^			
* BRCA1*	55 (58)	27 (57)	28 (58)
* BRCA2*	40 (42)	20 (43)	20 (42)
Age, mean (SD)^f^	41.37 (11.06)	44.06 (13.05)	38.68 (7.87)
Time since variant discovery, mean (SD), y	4.7 (3.32)	5.071 (3.88)	4.33 (2.65)
Genetic testing initiative^c^			
Self	32 (32)	15 (31)	17 (34)
Family	46 (47)	22 (45)	24 (48)
Physician	15 (15)	6 (12)	9 (18)
Other	6 (6)	6 (12)	0
Testing status among family members			
Yes	95 (95)	47 (94)	48 (96)
No	4 (4)	2 (4)	2 (4)
Unknown	1 (1)	1 (2)	0
Family history of breast or ovarian cancer			
Yes	91 (91)	44 (88)	47 (94)
No	9 (9)	6 (12)	3 (6)
Family history of other cancers			
Yes	74 (74)	37 (74)	37 (74)
No	23 (23)	11 (22)	12 (24)
Unknown	3 (3)	2 (4)	1 (2)

Abbreviation: IBSR, inquiry-based stress reduction.

^a^ Other countries of birth include 2 women from Ukraine, 2 women from the United Kingdom, 1 woman from Uruguay, 1 woman from the United States, 1 woman from Romania, and 2 women from other countries not specified.

^b^ Includes 7 single women, 13 divorced women, and 1 widowed woman.

^c^ Includes responses from 99 women (49 women in the IBSR group and 50 women in the control group).

^d^ Religious indicates orthodox Jewish; traditional, Masorti Jewish.

^e^ Includes responses from 95 women (47 women in the IBSR group and 48 women in the control group).

^f^ Two participants in the control group who were nearly 70 years old contributed to age differences between groups. Without these 2 participants, no significant difference would have been observed between groups.

### Psychological Well-being

There were no significant differences found at baseline between the IBSR and control groups for any of the psychological well-being outcomes except for personal growth subscale (autonomy: 55.20 [11.12] vs 56.77 [9.90]; environmental control: 56.30 [11.98 vs 58.51 [11.41]; personal growth: 63.70 [14.66] vs 68.85 [8.07]; positive relationships: 63.10 [15.91] vs 68.10 [9.86]; goals in life: 60.00 [14.12] vs 64.82 [10.57]; self-acceptance: 55.02 [16.62] vs 60.32 [13.50]) (Table 2). At T2, statistically significant differences between the IBSR and control groups were noted in mean (SD) scores for all 6 dimensions of psychological well-being, including self-acceptance (66.93 [11.15] vs 58.09 [15.55]; *P* < .001), positive relationships with others (71.24 [9.99] vs 65.06 [12.58]; *P* < .001), autonomy (63.64 [8.35] vs 54.73 [10.41]; *P* < .001), environmental control (63.95 [10.05] vs 57.45 [11.43]; *P* < .001), personal growth (73.00 [8.34] vs 65.76 [10.95]; *P* < .001), and goals in life (67.57 [8.88] vs 61.18 [12.87]; *P* < .001) (Table 2). At T3, the IBSR group continued to experience improved mean (SD) scores compared with the control group (autonomy: 62.68 [9.05] vs 56.12 [10.64]; environmental control: 64.55 [10.28] vs 59.35 [12.98]; personal growth: 72.00 [8.06] vs 67.15 [11.82]; positive relationships: 71.24 [9.78] vs 66.92 [12.37]; goals in life: 68.33 [8.54] vs 62.92 [13.24]; self-acceptance: 66.84 [11.35] vs 58.97 [17.03]). Sensitivity analysis did not show any interaction of age on differences between treatment groups or measurement occasion in the primary outcome.

**Table 2.** Effect of Intervention on Psychological Well-being

Measure	Group				Time × group interaction (between groups), *P* value^b^
	IBSR		Control		
	Mean (SD)	*P* value^a^	Mean (SD)	*P* value^a^	
Autonomy					
T1	55.20 (11.12)	NA	56.77 (9.90)	NA	.51
T2	63.64 (8.35)	.001	54.73 (10.41)	.18	<.001
T3	62.68 (9.05)	<.001	56.12 (10.64)	.88	<.001
Environmental control					
T1	56.30 (11.98)	NA	58.51 (11.41)	NA	.39
T2	63.95 (10.05)	.001	57.45 (11.43)	.048	<.001
T3	64.55 (10.28)	<.001	59.35 (12.98)	.34	<.001
Personal Growth					
T1	63.70 (14.66)	NA	68.85 (8.07)	NA	.03
T2	73.00 (8.34)	.001	65.76 (10.95)	.048	<.001
T3	72.00 (8.06)	<.001	67.15 (11.82)	.20	<.001
Positive relationships with others					
T1	63.10 (15.91)	NA	68.10 (9.86)	NA	.07
T2	71.17 (9.99)	.04	65.06 (12.58)	.03	<.001
T3	71.24 (9.78)	<.001	66.92 (12.37)	.35	
Goals in Life					
T1	60.00 (14.12)	NA	64.82 (10.57)	NA	.07
T2	67.57 (8.88)	.001	61.18 (12.87)	.05	<.001
T3	68.33 (8.54)	<.001	62.92 (13.24)	.18	<.001
Self-acceptance					
T1	55.02 (16.62)	NA	60.32 (13.50)	NA	.11
T2	66.93 (11.15)	.001	58.09 (15.55)	.11	<.001
T3	66.84 (11.35)	<.001	58.97 (17.03)	.23	<.001

Abbreviations: IBSR, inquiry-based stress reduction; NA, not applicable; T1, baseline; T2, immediately after the intervention; T3, 12-week follow-up.

^a^ *P* values are vs T1.

^b^ A mixed-models analysis was used to measure interactions between time variables and group. The dependent variable was the psychological well-being variable, and the independent variables were time (T1, T2, and T3), group (IBSR and control), and interaction between the time × group. Sensitivity analysis used a mixed-effects model with age, as there were differences in T1 data between the groups. Multiple comparisons were made according to the Hochberg Binyamini model.

### Secondary Outcomes

#### Sleep Quality

There was no statistically significant difference between groups in secondary outcomes at baseline (Table 3 and Table 4). We noted significant positive effects of the intervention on the participants’ sleep quality (Table 3). Mean scores in the intervention group, compared with controls, were improved at T3: sleep quality in the intervention group was clinically improved: from poor sleep quality (mean [SD], 7.35 [3.97]) at T1 to normal quality (mean [SD], 4.63 [3.21]; *P* < .001) at T3, as indicated by values less than 5. In the control group, the quality of sleep remained poor (mean [SD]: T1, 7.17 [4.45]; T3, 6.80 [3.66]; *P* = .64).

**Table 3.** Effect of IBSR on Sleep Quality in Women With *BRCA* Variants

Variable	Time	Group				*P* value, time × group interaction (between groups)^b^
		IBSR		Control		
		Mean (SD)	*P* value^a^	Mean (SD)	*P* value^a^	
Sleep quality^c^	T1	7.35 (3.97)	NA	7.17 (4.45)	NA	.84
	T2	4.76 (3.76)	<.001	6.72 (3.57)	.57	.06
	T3	4.63 (3.21)	<.001	6.80 (3.66)	.64	.04

Abbreviations: IBSR, inquiry-based stress reduction; NA, not applicable; T1, baseline; T2, immediately after the intervention; T3, 12-week follow-up.

^a^ *P* values are vs T1.

^b^ A mixed-models analysis was used to measure interactions between time variables and group. The dependent variable was the psychological well-being variable, and the independent variables were time (T1, T2, and T3), group (IBSR and control), and interaction between the time × group. Sensitivity analysis used a mixed-effects model with age, as there were differences in T1 data between the groups. Multiple comparisons were made according to the Hochberg Binyamini model.

^c^ Assessed using the Pittsburgh Sleep Quality Inventory. Scores of 5 or greater are considered poor-quality sleep.

**Table 4.** Perceptions of Risk-Reducing Surgical Procedures

Perception on procedure	Participants, No. (%)		
	T1	T2	T3
**Prophylactic oophorectomy**			
Control			
Out of the question^a^	2 (4)	2 (5)	3 (8)
Considering^b^	26 (52)	20 (46)	21 (53)
In favor of procedure^c^	22 (44)	21 (49)	16 (40)
IBSR			
Out of the question^a^	7 (14)	2 (4)	1 (2)
Considering^b^	24 (48)	24 (50)	16 (35)
In favor of procedure^c^	19 (38)	22 (46)	29 (63)
Total			
Out of the question^a^	9 (9)	4 (4)	4 (5)
Considering^b^	50 (50)	44 (48)	37 (43)
In favor of procedure^c^	41 (41)	43 (47)	45 (52)
Between-group *P* value	.048^d^	.07^e^	.04^f^
**Prophylactic mastectomy**			
Control			
Out of the question^a^	17 (34)	20 (49)	21 (54)
Considering^b^	25 (50)	11 (27)	13 (33)
In favor of procedure^c^	8 (16)	10 (24)	5 (13)
IBSR			
Out of the question^a^	23 (46)	22 (47)	13 (29)
Considering^b^	21 (42)	19 (40)	17 (39)
In favor of procedure^c^	6 (12)	6 (13)	14 (32)
Total			
Out of the question^a^	40 (40)	42 (48)	34 (41)
Considering^b^	46 (46)	30 (34)	30 (36)
In favor of procedure^c^	14 (14)	16 (18)	19 (23)
Between-group *P* value	<.001^d^	.004^e^	<.001^f^

Abbreviations: IBSR, inquiry-based stress reduction; T1, baseline; T2, immediately after the intervention; T3, 12-week follow-up.

^a^ The full response was given as *the surgery is out of the question, or I may consider it only if I discover a tumor* (in Hebrew).

^b^ The full response was *I am considering the surgery, but I haven’t made a final decision, or I am waiting until my childbearing years are over* (in Hebrew).

^c^ The full response was *I have made an appointment or have already had the surgery* (in Hebrew)*.*

^d^ Between-group comparison of T1 vs T2.

^e^ Between-group comparison of T2 vs T3.

^f^ Between-group comparison of T1 vs T3.

### Psychosocial Variables

An exploratory analysis indicated improvements in the intervention group in positive emotions (mean [SD] score: T1, 25.96 [6.87]; T3, 27.82 [10.51]; *P* = .02) and general self-ability (mean [SD] score: T1, 29.46 [6.31]; T3, 33.18 [5.03]; *P* = .004). No differences were noted between the IBSR and control groups in optimism, health perception, mindfulness, or life satisfaction (eFigure in Supplement 2).

### Views on Risk-Reducing Surgical Procedures

The participants were queried about their intentions and attitudes regarding risk-reducing surgical procedures: bilateral mastectomy and/or salpingo-oophorectomy. The recommendations for risk-reducing surgical procedures were offered by clinicians to all study participants. At T1, salpingo-oophorectomy was not a viable option for the smallest proportion of participants (total: 9 participants [9%]; IBSR: 7 participants [14%]). After the intervention, only 1 participant (2%) from the IBSR group remained opposed to risk-reducing surgery (*P* = .04). In the IBSR group, 2 trends were observed regarding risk-reducing mastectomy: the number of women who underwent mastectomy or made an appointment to gain more professional insight into this the possibility went up from 6 participants (12%) to 14 participants (31%) (*P* < .001), while the number of women for whom risk-reducing mastectomy was not even considered as an option decreased from 23 participants (46%) to 13 participants (29%) (*P* < .001).

## Discussion

This randomized clinical trial is the largest adequately powered trial to date, to our knowledge, that tested the effect of a psychosocial intervention on psychological well-being and health choices in asymptomatic women with *BRCA* variants using validated data tools. The results show that an IBSR-based intervention improved well-being and sleep-quality parameters compared with usual care. The improvement was sustained for at least 3 months after the end of the intervention. Additionally, the participants in the intervention group were less reluctant to consider risk-reducing surgical procedures.

Individuals with *BRCA* variants may experience disturbances in psychological well-being as a result of the substantially higher lifetime risk for developing cancer.^6^ Yet, it has been shown that it is possible to improve well-being using behavioral interventions.^25^ In concordance with these findings, our 3-month long intervention involving the IBSR technique caused a sustained improvement in all 6 measures of psychological well-being (self-acceptance, positive relations with others, autonomy, environmental mastery, purpose in life, and personal growth) compared with adequately matched controls. This stress-reduction technique is a self-practice tool that can be continually used by women on their own, in conjunction with genetic consultation and the advised standard of care.

Poor-quality sleeping patterns have been identified as significant risk factors associated with morbidity and mortality in prospective population studies.^50^ Shorter-than-advised sleep is associated with an increased risk for developing diabetes, hypertension, cardiovascular diseases, coronary heart diseases, and obesity,^51^ with some studies also reporting associations with mood disturbances, anxiety symptoms, and depression.^52^ Poor-quality sleeping patterns have been documented in women *BRCA* variants and were associated with fatigue, cancer-related worry, and psychological distress.^12^ In this study, a significant improvement and return to the normal range of sleep quality were noted in the intervention group compared with the control group. These findings are consistent with those reported in a previous study on IBSR, which focused on women in Israel who survived breast cancer.^32^

Studies have reported that general self-efficacy is associated with enhanced mental and physical health, since it may encourage health-related behaviors.^53^ A group-based patient education intervention was studied in Norway between 2011 and 2013 and found that women with newly diagnosed *BRCA* variants reported lower self-efficacy levels and were more vulnerable to stress and anxiety.^9^ Our exploratory results demonstrated an improvement in general self-efficacy in the intervention group compared with the control group. Given this empirical evidence, it is plausible that women in the intervention group who practiced IBSR and significantly enhanced their general self-efficacy would cope better with stressful events or emotional distress related to their carrier status. Our exploratory findings showed an increase in positive emotions and decrease in negative emotions in the intervention group compared with the control group. A careful literature search yielded no other studies that analyzed the associations of psychosocial interventions with positive and negative emotions in women with *BRCA* variants. Since emotional distress among patients seeking genetic counseling for hereditary cancer is common,^8,9^ further studies are recommended to address the role of psychosocial interventions in supporting emotional distress of women with *BRCA* variants. We did not find differences in other variables between the study groups.

The only proven active risk-reduction procedures currently available and offered to women with *BRCA* variants are surgical: bilateral mastectomy and/or salpingo-oophorectomy.^54^ In Israel, only 10% to 13% of asymptomatic women with *BRCA* variants opt for a risk-reducing mastectomy.^54^ A 2019 study that assessed cancer screening and risk-reduction options in a cohort of 6223 women with *BRCA* variants from 10 countries found that 27.8% underwent bilateral risk-reducing mastectomy, and 64.7% had a risk-reducing salpingo-oophorectomy.^55^ The reasons for avoiding the surgical procedure options include fear of surgery and its possible complications,^56,57^ risk of early menopause symptoms (eg, depression, anxiety),^58^ and its impact on the quality of life, body image, and sexual functions, among others.^58,59^ In our study, all parameters assessing attitudes and acceptance of risk-reducing surgical procedures and taking concrete steps for performing these surgical procedures improved in the IBSR group. Thus, it appears that an IBSR intervention may support women with *BRCA* variants in adopting a more favorable view on the possibility of undergoing risk-reducing procedures.

### Limitations

This study has several limitations. The study was conducted in a research hospital, with a relatively small sample size that included Jewish participants with high socioeconomic status. While this reflects the sample composition of study participants at our institution, future studies should include ethnically and socioeconomically diverse samples of women with *BRCA* variants. Second, after the completion of the IBSR workshop during weeks 12 to 24 of the follow-up period, there was no record kept of the time spent practicing IBSR. Future research should include the measurement of longer-term follow-ups to determine the duration of the effects of the IBSR intervention. Third, a self-report is prone to the social desirability bias, which we attempted to mitigate through the collection of anonymized data. Fourth, owing to the nature of the intervention, participants were aware of group allocation for the duration of the study. To reduce to reduce the risk of bias, participants were informed of their allocation only after completion of the baseline questionnaire, and the clinicians were blinded to the allocation of participants to either the treatment or control group.

## Conclusions

This randomized clinical trial found that an IBSR intervention in asymptomatic women with pathogenic *BRCA* variants improved well-being and sleep-quality and led to a more favorable attitude toward risk-reducing surgical procedures in the intervention group compared with control group. IBSR may be implemented as a self-practice tool to enhance well-being among women with *BRCA* variants and support them in their treatment decision-making processes.

## Notes

Supplement 1.
Trial Protocol and Statistical Analysis Plan

Supplement 2.
eMethods.
**eFigure.** IBSR Intervention Worksheet: Judge-Your-Neighbor Worksheet **eTable.** Effect of the Intervention on Psychosocial Variables: Exploratory Analysis

Supplement 3.
Data Sharing Statement
