# Supplementary material for: Effect of Inquiry-Based Stress Reduction on Well-being and Views on Risk-Reducing Surgery Among Women With BRCA Variants in Israel: A Randomized Clinical Trial
Source: JAMA Netw Open. 2021 Dec 28;4(12):e2139670. doi: 10.1001/jamanetworkopen.2021.39670 (PMC8715352; doi:10.1001/jamanetworkopen.2021.39670)
Supplement: Supplement 1. — Trial Protocol and Statistical Analysis Plan [file jamanetwopen-e2139670-s001.pdf]

Study protocol

Effect of Inquiry Based Stress Reduction (IBSR) on well-being and views on risk-reducing surgery among Israeli female BRCA1/BRCA2 mutation carriers: a randomized controlled trial.

Date: July 1, 2017 - October 1, 2020

Sponsor: Sheba Medical Center

Investigators

Principal Investigator:	Eitan Friedman, Prof.	Sheba Medical Center
Principal Investigator:	Shahar Lev- Ari, Dr.	Tel Aviv University
Study coordinator:	Carla Landau.	Tel Aviv University

Summary:

Breast cancer (BC) is a major health problem and the most prevalent cancer among women.In a substantial proportion of familial cases, germ-line mutations in either BRCA1/2 can be detected. The only proven modality for active risk reduction (rather than passive early detection), is prophylactic surgery - prophylactic mastectomy and oophorectomy. While the majority of Jewish mutation carriers elect to undergo prophylactic oophorectomy at about age 40 years, in Israel only a minority perform prophylactic mastectomy. Another ramification of being a mutation carrier is the emotional stress associated with that discovery.Genetic information has profound implications for mutation carriers. The IBSR (Inquiry-based stress reduction) intervention, developed by Byron Katie, trains participants to reduce their perceived level of stress by self-inquiry of their thoughts and beliefs connected to stressful circumstances or symptoms. This meditative process, named "The Work", enables the participants to identify and question the stressful thoughts that cause their suffering. The core of IBSR is simply four questions and a turnaround, which is a way of experiencing the opposite of what the participant believes. This process is simple, powerful and provides skills for self-inquiry and management of stressful thoughts that can be easily implemented in daily life. Therefore, on the basis of previous data and beneficial observations we postulate that the clinical utility of IBSR mediation program may improve psychological and physical symptoms and quality of life among asymptomatic (oncologically healthy) BRCA1/ BRCA2 mutation carriers. Thus, we will conduct a pilot randomized controlled trial to scientifically investigate the effect of this intervention effects on BRCA1/2 mutation carriers.

Study design:

Allocation: Randomized
Intervention Model: Parallel Assignment

Masking: None (Open Label)
Primary Purpose: Treatment

Arms and interventions:

- Experimental: Intervention

  Inquiry Based Stress Reduction

  Intervention: Behavioral: Inquiry Based Stress Reduction (IBSR) program

  Placebo Comparator: Control

  The placebo group participants will receive book of IBSR intervention at the close of the study.

  Intervention: Health Promotion–lifestyle modification: Control

Primary Outcome measures:

The Ryff scale- Psychological well-being- quantified using the Ryff questionnaire (84-item). includes six subscales that evaluate: self-acceptance, positive relationships with others, autonomy, control of the environment, personal growth, and goals in life. For each of the dimensions, the score ranges from 14 and 84. Higher scores indicate higher psychological well-being [33].

Secondary Outcome measures:

- **Sleep Quality** - assessed using the Pittsburgh Sleep Quality Inventory Questionnaire (PSQI)[33,34] 19-items (score range 0-21) for self-reporting of sleep quality over one month.
- **Psychosocial variables (exploratory)**:
  - positive and negative emotions (PANAS)[35], a 20-item questionnaire (score range 10-50 for each subscale) that measures the emotional positive and negative state of individuals;
  - Satisfaction with Life Scale (SWLS)[36], 5-item scale (score range 7-35) that measures global life satisfaction; self-assessed health (SAH)[37], a single question designed to assess how the participant evaluates their health;
  - Perceived Social Support from Family (PSSFA)[38], 20-item scale (score range 0-20) that assess the family support as perceived by the respondents;
  - mindfulness (MAAS)[39], 15-item scale (score range 15-90) that measure level of attention and mindfulness to the present;
  - Life Orientation Test-Revised (LOT-R)[40], 10-item questionnaire (score range 0-24) that measures optimism as a character trait, expected to remain stable throughout one's lifetime
  - General self-ability (GSE)[41], 10-item scale that measures the general perception of personal agency in order to forecast the individual's ability to cope with everyday distresses and assess their adaptation to stressful events.

- **Health behaviors**: evaluated by the attitudes towards and actual performance of risk reduction surgical procedures of the breasts and/or fallopian tubes and ovaries[42].

Eligibility criteria
Ages: 25 to 70.
Sexes: female.

- Inclusion criteria: Women who are carriers of one of the predominant Jewish mutations in BRCA1/2 genes, age 25-70 years, with no current or past history of cancer (except BCC), who are willing to sign an informed consent, and present mental clarity by the ability to comprehend and fulfill all the questionnaires.
- Exclusion criteria: Diagnosed with breast or ovarian cancer or any other cancer, risk reduction mastectomy, severe psychiatric diagnosis (e.g. bipolar disorder).

Statistical Plan:

An intention-to-treat analysis approach will be implemented for this trial, and all subjects will be included in the data processing. The similarity of baseline (T1) demographic characteristics of participants in the control and intervention groups will be assessed using the Chi-squared test for categorical variables and independent samples t-test for continuous variables. Differences between the groups will be tested using mixed model analysis over the three different time points as well as the interaction between group and time. The dependent variables is the study outcomes, and the independent variables are time (before, immediately post-intervention, and during the follow-up period), group (intervention and control), and interaction between the time X the group. The difference between the outcome variables will be measured using the entry values (time point 1) and the final values gathered at the end of the follow-up (time point 3). Control for multiple comparisons will be done using the False Discovery Rate approach. An analysis of the research hypotheses of the categorical variables and variables without normal distribution will be performed according to the General Estimated Equation (GEE). To check the internal consistency of the research tools, an alpha Cronbach coefficient test will be performed for each tool. The statistical software SPSS 26 (IBM, New York City) will be be used independently for all the data processing within this study by two researchers.
