# Supplementary material for: Effect of Inquiry-Based Stress Reduction on Well-being and Views on Risk-Reducing Surgery Among Women With BRCA Variants in Israel: A Randomized Clinical Trial
Source: JAMA Netw Open. 2021 Dec 28;4(12):e2139670. doi: 10.1001/jamanetworkopen.2021.39670 (PMC8715352; doi:10.1001/jamanetworkopen.2021.39670)
Supplement: Supplement 2. — eMethods. eFigure. IBSR Intervention Worksheet: Judge-Your-Neighbor Worksheet eTable. Effect of the Intervention on Psychosocial Variables: Exploratory Analysis [file jamanetwopen-e2139670-s002.pdf]

Supplemental Online Content

Landau C, Novak AM, Ganz AB, Rolnik B, Friedman E, Lev-Ari S. Effect of inquiry-based stress reduction on well-being and views on risk-reducing surgery among women with *BRCA* variants in Israel: a randomized clinical trial. *JAMA Netw Open*. 2021;4(12):e2139670. doi:10.1001/jamanetworkopen.2021.39670

eMethods.
eFigure. IBSR Intervention Worksheet: Judge-Your-Neighbor Worksheet
eTable. Effect of the Intervention on Psychosocial Variables: Exploratory Analysis

This supplemental material has been provided by the authors to give readers additional information about their work.

This supplemental material has been provided by the authors to give readers additional information about their work.

eMethods

Cronbach's Alpha for instruments

Instrument	Time	Cronbach's Alpha
Ryff's Psychological Well-being Scales, Dimension 1: **Autonomy**	T1	0.83
Ryff's Psychological Well-being Scales, Dimension 2: **Environmental control**	T1	0.84
Ryff's Psychological Well-being Scales, Dimension 3: **Personal growth**	T1	0.89
Ryff's Psychological Well-being Scales, Dimension 4: **Positive relationships with others**	T1	0.84
Ryff's Psychological Well-being Scales, Dimension 5: **Goals in life**	T1	0.89
Ryff's Psychological Well-being Scales, Dimension 6: **Self-acceptance**	T1	0.93
Life Orientation Test – Revised (LOT-R) **Systemic Optimism**	T1	0.80
Pittsburgh Sleep Quality Inventory Questionnaire (PSQI) **Sleep quality**	T1	0.73
Positive and Negative Affect Schedule Scale (PANAS) **Positive emotions**	T1	0.90
Positive and Negative Affect Schedule Scale (PANAS) **Negative emotions**	T1	0.90
Perceived Social Support from Family (PSSFA) **Perception of family support**	T1	0.90
The Mindfulness Attention Awareness Scale (MAAS) **Mindfulness**	T1	0.89
Satisfaction with Life Scale (SWLS) **Life satisfaction**	T1	0.93
General Self-ability Scale (GSE) **General self-ability**	T1	0.92

© 2021 Landau C et al. *JAMA Network Open.*

eFigure. IBSR Intervention Worksheet: Judge-Your-Neighbor Worksheet

Judge-Your-Neighbor Worksheet

Think of a stressful situation with someone—for example, an argument. As you meditate on that specific time and place and begin to feel what that felt like, fill in the blanks below. Use short, simple sentences.

1. In this situation, who angers, confuses, hurts, saddens, or disappoints you, and why?

I am ____________ (emotion) with ____________ (name) because ____________________________

I am angry with Paul because he lied to me.

WANTS 2. In this situation, how do you want him/her to change? What do you want him/her to do?

I want ____________ (name) to ____________________________

I want Paul to see that he is wrong. I want him to stop lying to me.

ADVICE 3. In this situation, what advice would you offer him/her? "He/she should/shouldn't..."

____________ (name) should/shouldn't ____________________________

Paul shouldn't frighten me with his behavior. He should take a deep breath.

NEEDS 4. In order for *you* to be happy in this situation, what do you need him/her to think, say, feel, or do?

I need ____________ (name) to ____________________________

I need Paul to stop talking over me. I need him to really listen to me.

COMPLAINTS 5. What do you think of him/her in this situation? Make a list. (It's okay to be petty and judgmental.)

____________ (name) is ____________________________

Paul is a liar, arrogant, loud, dishonest, and unconscious.

6. What is it about this person and situation that you don't ever want to experience again?

I don't ever want ____________________________

I don't ever want Paul to lie to me again. I don't ever want to be disrespected again.

Now question each of your statements, using the four questions of The Work, below. For the turnaround to statement 6, replace the words *I don't ever want*... with *I am willing to*... and *I look forward to*...

The four questions

Example: Paul lied to me.

1. Is it true? (Yes or no. If no, move to question 3.)
2. Can you absolutely know that it's true? (Yes or no.)
3. How do you react, what happens, when you believe that thought?
4. Who or what would you be without the thought?

Turn the thought around.

I lied to me.

I lied to *Paul.*

Paul *didn't* lie to me.

Paul *told me the truth.*

As you visualize the situation, contemplate how each turnaround is as true or truer.

THE WORK OF BYRON KATIE © 2019 Byron Katie International, Inc. All rights reserved. thework.com

6Feb. 2019

eTable. Effect of the Intervention on Psychosocial Variables: Exploratory Analysis

Variable	Time	Group						Time × group interaction (between groups)*
		Intervention			Control			
		Mean	SD	*P* Value	Mean	SD	*P* Value	*P* Value
Systemic optimism Lot-R	T1	12.54	2.77	-	12.75	3.71	-	0.909
	T2	14.26	4.09	-	13.39	4.04	-	
	T3	14.02	3.7	-	14.15	4.75	-	
	T1-T2			0.006			0.265	0.481
	T2-T3			0.745			0.302	0.332
Positive emotions PANAS	T1	25.96	6.87	-	27.68	8.44	0.033	0.007*
	T2	28.75	9.12	-	24.25	8.81	0.302	
	T3	27.82	10.51	-	24.04	6.50	-	
	T1-T2			0.022			0.035	0.003*
	T2-T3			0.53			0.925	0.713
Negative Emotions PANAS	T1	34.47	8.37	-	34.06	9.82	-	0.027*
	T2	32.22	9.98	-	37.19	7.38	-	
	T3	33.46	9.63	-	38.44	7.70	-	
	T1-T2			0.162			0.069	0.030*
	T2-T3			0.447			0.484	0.979
Perception of family support PSS-FA	T1	16.38	4.81	-	16.6	4.02	-	0.486
	T2	16.85	3.43	-	16.58	3.98	-	
	T3	17.26	3.26	-	18.26	3.13	-	
	T1-T2			0.542			0.978	0.534
	T2-T3			0.609			0.043	0.265
Mindfulness MAAS	T1	3.97	0.77	-	3.93	0.88	-	0.692
	T2	4.18	0.96	-	4.14	0.84	-	
	T3	4.47	0.97	-	4.30	0.66	-	
	T1-T2			0.351			0.235	0.755
	T2-T3			0.045			0.374	0.467
Life Satisfaction SWLS	T1	23.48	6.47	-	22.6	6.19	-	0.65
	T2	25.86	5.72	-	25.21	6.4	-	
	T3	25.75	6.03	-	26.4	5.69	-	
	T1-T2			0.039			0.034	0.89
	T2-T3			0.946			0.319	0.444
General self-efficacy GSE	T1	29.46	6.31	-	30.68	5.44	-	0.004*
	T2	32.75	4.26	-	31.4	6.3	-	
	T3	33.18	5.03	-	30.79	6.35	-	
	T1-T2			0.004			0.526	0.158

© 2021 Landau C et al. *JAMA Network Open.*

	T2-T3			0.701			0.604	0.523
Health Perception SAH	T1	2.54	0.95	-	2.26	0.66	-	0.925
	T2	1.98	0.76	-	2.09	0.92	-	
	T3	2.06	0.82	-	2	0.87	-	
	T1-T2			0.02			0.097	0.089
	T2-T3			0.622			0.592	0.389

was performed using a mixed effects model was used to measure interactions between time and group variables The dependent variable was psycho-social variable, and the independent variables were time (before ,after intervention, and follow-up period), group (intervention and control), and interaction between time and group. Sensitivity analysis was conducted using a mixed-effects model with age due to differences in age at baseline between the groups. Exploratory analysis: *P<0.05.

© 2021 Landau C et al. *JAMA Network Open.*
