# Supplementary material for: Effect of Inquiry-Based Stress Reduction on Well-being and Views on Risk-Reducing Surgery Among Women With BRCA Variants in Israel: A Randomized Clinical Trial
Source: JAMA Netw Open. 2021 Dec 28;4(12):e2139670. doi: 10.1001/jamanetworkopen.2021.39670 (PMC8715352; doi:10.1001/jamanetworkopen.2021.39670)
Supplement: Supplement 3. — Data Sharing Statement [file jamanetwopen-e2139670-s003.pdf]

Data Sharing Statement

Landau. Effect of Inquiry-Based Stress Reduction on Well-being and Views on Risk-Reducing Surgery Among Women With BRCA Variants in Israel. *JAMA Netw Open*. Published December 28, 2021. doi:10.1001/jamanetworkopen.2021.39670

Data
Data available: No

Additional Information
Explanation for why data not available: The data presented herein cannot be made public nor can these be shared by others, as per the regulations of the ethics committee that approved this study
